# Neurokinin B and pre-eclampsia: a decade of discovery

**DOI:** 10.1186/1477-7827-8-4

**Published:** 2010-01-14

**Authors:** Nigel M Page

**Affiliations:** 1 School of Life Sciences, Kingston University London, Penrhyn Road, Kingston-upon-Thames, Surrey, KT1 2EE, UK

## Abstract

At the start of the last decade, we provided evidence that levels of the peptide neurokinin B were highly elevated in pre-eclampsia. We hypothesized that elevated levels of neurokinin B may be an indicator of pre-eclampsia and that treatment with certain neurokinin receptor antagonists may be useful in alleviating the symptoms. At the time of the original hypothesis many questions remained outstanding. These included - Does neurokinin B have any diagnostic value in the detection and diagnosis of pre-eclampsia? - What is the cause of the elevated levels of neurokinin B during pre-eclampsia? - What is the physiological significance of neurokinin B in the placenta? This review discusses the answers to these questions taking into account the subsequent developments of the past ten years and analyzing the plethora of discoveries that have arisen from those initial observations.

## Background

The precise origin of pre-eclampsia remains elusive, but it is believed to be multifactorial and to include a scenario of required steps [1]. One crucial step is defective trophoblast invasion, which is thought to lead to reduced placental perfusion. We have believed, like many others that a poorly diffused and ischaemic placenta releases an excess of a factor(s) and, it is this factor(s) that leads to the cascade of events defined as the clinical syndrome of pre-eclampsia. Roberts [2] predicted that any candidate molecule(s) would not be unique but rather a known molecule(s) that is present in excessive amounts. In addition, any factor is likely to be placental in origin as it is accepted that it is the presence of the placenta rather than the fetus, which is responsible for the development of pre-eclampsia [3]. In the late 1990s we sought to identify potential secreted factors from the placenta using mRNA fingerprinting [4] and the human genomic databases [5] and found nine matches that showed high similarity to the bovine neurokinin B (NKB) precursor [6]. In all, we identified more than 2,000 sequences of known genes; expressed sequence tags and uncharacterized genes. These genes have also included variants of pregnancy associated plasma protein-E [7], the apolipoprotein L proteins [8] and the tachykinin gene-related peptides [9].

From all the candidates NKB, which is a member of the tachykinin family [10], appeared to be the most promising as a potential marker and factor to cause pre-eclampsia. The placenta was found to express unusually high levels of *TAC*3 that encodes NKB [11], a gene previously believed not to be expressed in any peripheral tissue [12]. This expression was located to the outer syncytiotrophoblasts in an ideal position for secretion into the maternal blood [6]. In translational terms significant amounts of immunoreactive human NKB were found in early and term placenta, and maternal plasma, which had identical chromatographic properties to synthetic NKB [6,13]. Using a commercial radioimmunoassay (RIA), NKB plasma concentrations were found to be low or undetectable from normotensive pregnancies although a proportion did exhibit a slight rise at term. Four normotensive pregnancies between weeks 9 and 14 also had concentrations equivalent to the highest at term. From eight pre-eclamptic women tested in their third trimester all were found to have considerably higher levels of NKB [6]. NKB was also found in cord blood indicating that the secreted peptide could also enter and affect the feto-placental circulation, however, its role in the fetus is unknown [6]. In terms of a marker, placental NKB appeared to have several advantages over other candidate markers as it appeared not only unique to pre-eclampsia but also to pregnancy and was not associated with other known hypertensive disorders [5]. In addition, evidence from the infusion of high doses of NKB into female rats indicated that NKB might be involved in some of the haemodynamic events of pre-eclampsia [6,11]. Here, NKB was found to cause substantial pressor activity (believed initiated through the NKB preferred receptor NK3) and a 37% gain in uterine weight providing indirect evidence for an increase in blood supply to the uterus [6]. We, therefore, proposed that NKB could be a signal sent from the placenta to the mother in order to maximize blood flow to the feto-placental unit. It is also possible for NKB to progressively stimulate other NK receptors (NK1 and NK2) and cause the additional clinical features associated with pre-eclampsia such as thrombocytopenia [14], generalized inflammation [15], oedema [16] and eclampsia [17]. Nonetheless, at the time we proposed our original hypothesis many outstanding questions still remained. These included amongst others - does NKB have any diagnostic value in the detection and diagnosis of pre-eclampsia and what is the cause of the elevated levels of NKB during pre-eclampsia? What is the physiological significance of NKB in the placenta? We shall review the subsequent developments in each of these areas in turn.

## Does NKB have any diagnostic value?

In the case for a diagnostic marker the measurement of elevated levels of NKB in pregnancies complicated by pre-eclampsia has been subsequently confirmed in several studies [6,18-24]. Nevertheless, even though, all of these studies have shown elevated levels of NKB to be statistically significant, it perhaps should be noted that there are significant variations in the amounts of not only NKB measured from each of the different studies (some measured in the p/mol range, some n/mol, some ng/l and some μg/l), but in the fold differences observed (from 1.3 to 9 fold). Geissbuehler et al. [19] have suggested two reasons for this. The first involves the accuracy of NKB determination, which depends crucially on a reliable sample preparation. Plasma is used in preference to serum because the latter contains active proteases, which can affect the antigenicity of peptides. Currently, NKB levels cannot be measured directly in plasma and a lengthy and laborious acidified peptide extraction protocol is employed using Sep-Pak C18 cartridges, followed by peptide elution and vacuum centrifugation. For instance, Sakamoto et al. [25] calculated a mean recovery rate of NKB of only 58.4% using this method. The second reveals that there can be a high standard deviation in the pre-eclamptic groups, thereby reflecting a wider range of NKB levels measured in these women when compared to normotensive controls. This is not to be unexpected as the onset; severity and progression of pre-eclampsia can be significantly distinct between different women [26]. The type of immunoassay employed could also produce other differences. Traditionally, in the detection of NKB a commercial radioimmunoassay has been employed, but more recently this has been superseded by the utilisation of a commercial enzyme-linked immunosorbant assay (ELISA) [19,24,27]. Nonetheless, Geissbuehler et al. [19] found a high correlation between results of the RIA and ELISA in determining NKB concentrations in plasma supporting the effective use of both. We have also found comparable results between the RIA and ELISA when analysing placental chromatographic fractions. Nevertheless, although, the RIA does provide a highly sensitive method to measure NKB levels, the ELISA provides a more simplified protocol and can be completed on a single day rather than spread over 2 to 3 days, as is the case with the RIA. There are also issues in designing new NKB immunoassays. The current immunoassay format is based on a commercial one site immunoassay, however, particular difficulties can be encountered when trying to accommodate two immunoglobulins on such a short peptide; for instance when developing two site immunoassay formats. For example, it has been reported that when testing a two site immunoassay with 'in house' antibodies raised against the two halves of NKB (DMHDF and FVGLM-NH_2_) only trace amounts of NKB in the plasma from pre-eclamptic women and only minute amounts of NKB in placental extracts could be detected when compared to the results of the commercial RIA [28]. This briefly lead to a hypothesis that NKB in the placenta may not be amidated and instead may have the extended sequence of DMHDFFVGLMGKR (1.55 kDa) which perhaps only the RIA could detect [28]. However, it was noted at the time that this was unusual as antibodies raised against much longer COOH-terminally amidated peptides can be very specific for the COOH-amide and often do not recognize COOH extended forms of the same peptide. We accordingly tested the commercial antibody to determine whether it could recognize a non-amidated form of NKB using the synthetic peptide; glycine COOH-terminally extended NKB (DMHDFFVGLMG). This synthetic peptide showed no significant cross-reactivity with the commercial NKB antibody [24,27] indicating that amidation was a crucial part of the natural epitope recognized. In addition, we found that substance P (1.34 kDa) and a peptide (KRDMHDFFVGLMG, 1.55 kDa) with the same molecular mass as the proposed peptide (DMHDFFVGLMGKR) eluted 2 and 3 fractions ahead respectively of the NKB immunoreactive peak found in pre-eclamptic placenta extracts when using the commercial antibody on a Superdex^® ^Peptide HR 10/30 column [27]. This indicated that the proposed extended NKB peptide was significantly larger in molecular mass than could be actually detected in placenta immunologically. Therefore, in the short term, at least, the commercial one site RIA or ELISA assay appears the most effective method at detecting NKB in plasma and placenta samples.

In general, the differential diagnostic value of NKB has been determined predominantly around term, but does it have any predictive value early in pregnancy? We originally found low or undetectable levels of NKB throughout normotensive pregnancies though a proportion did exhibit higher levels during the first trimester comparable to levels found at term [6]. A few studies have consequently analysed NKB plasma levels during normal pregnancy [25,29,30] using longitudinal studies covering the period of weeks 8 to 40. D'Anna et al. [29] showed that there was a slight non-significant increase of NKB throughout the different stages of normal pregnancy. They concluded that their data was in accordance with the original findings [6] reporting the highest concentrations at term. Interestingly, their results also contained one to three women in each group of ten (weeks 11-13, 14-17, 22-26) who had levels of NKB substantially higher than the mean at 37-40 weeks although none of their women went on to develop pre-eclampsia. This perhaps indicated that they had identified pregnant women who were secreting 'corrective' levels of NKB into their maternal bloodstreams [11]. Sakamoto et al. [25] showed similar increasing levels of NKB, but did find these to be significant, between weeks 24-35 and 36-40, when compared to non-pregnant women. They also observed levels of NKB within 48 hours postpartum that were significantly lower than those found between 36-40 weeks of pregnancy. Taken together, these findings were interpreted that as placental mass increases there is a corresponding increase of NKB in the maternal bloodstream confirming the local production of NKB to be the placenta. More recently, Li et al. [22] have looked at the differences in NKB plasma levels in normotensive and pre-eclamptic pregnancies in a longitudinal study between weeks 10-14 and 30-34 of gestation. They found maternal plasma levels of NKB to be significantly increased in early pregnancy before the onset of clinical symptoms. This difference was unique to the pre-eclamptic group and was not significant between gestational hypertensive and normotensive pregnancies [22]. This is the first direct evidence that NKB could have a predictive value before the onset of clinical symptoms. In spite of this, an earlier study measuring NKB levels between weeks 10-20 of pregnancy in women who developed pre-eclampsia or delivered growth restricted babies found no difference in NKB plasma levels [30]. Other interesting results have also become apparent with one study confirming elevated levels of NKB in pre-eclamptic women that could be split into two cohorts those with higher and lower concentrations of NKB [19]. The only clinical difference between the groups was that gravidity and parity were both higher in the high NKB cohort. This could perhaps indicate a more adverse reaction following sensitization to a first pregnancy. Differences in NKB levels between ethnic groups with pre-eclampsia have also been observed between those of a 'mixed ancestry' presenting with substantially higher levels of plasma NKB from those of a 'black ancestry' [21]. These variations occurred even though no significant differences in maternal clinical data were found between the two ethnic groups. Furthermore, NKB could possibly be used to differentiate between mild and severe pre-eclampsia. Immunostaining of human placental samples with a monoclonal NKB antibody revealed quantifiable levels of NKB immunoreactive staining of which those with mild (P > 0.05) or severe pre-eclampsia (P > 0.01) had significantly higher NKB staining levels than women in the control group [23].

It would seem apparent that NKB could have a diagnostic role to play at term and could also serve a role in determining differences between those with differing severities of the disorder. This may serve to allow some degree of sorting of pregnant women into different categories that can then be used to improve their clinical management. Its role as a predictor in earlier pregnancy before the onset of clinical symptoms is less clear. Although Li et al. [22] have confirmed predictive levels as early as weeks 10-14, other studies suggest that higher levels could be secreted in some patients who subsequently do not go on to develop pre-eclampsia. Further longitudinal studies are required to ascertain the predictive value of NKB and to determine at what gestational age it may be useful. NKB will have a more significant predictive value to the obstetrician if it can predict pre-eclampsia before the onset of symptoms. Regrettably, the current protocols for testing still involve a tedious extracted plasma assay and there does not seem to be a more user friendly and robust microtitre plate assay on the horizon quite yet. The detection of high levels of NKB remains unique to pregnancy and has not yet been associated with any other hypertensive disorders. However, high levels of NKB have recently been associated with other gestational diseases including intrauterine growth restriction (IUGR) [18] and pre-term labour [31] all of which can co-present with pre-eclampsia. NKB may therefore be able to provide an indication to the level of placental dysfunction.

## What is the cause of the elevated levels of NKB during pre-eclampsia?

Defective trophoblast invasion has been described as a consistent feature of pre-eclampsia that can lead to placental ischaemia and the potential release of NKB as a signal to the mother to maximize blood flow to the feto-placental unit. Proposed physiological triggers include hypoxia [32] and oxidative stress [33] and these have been shown to have an effect on the expression of a range of placental factors. Elevated expression levels of *TAC*3 are found to be significantly higher in pre-eclamptic placenta at term when compared to controls [22,34]; a result consistent with the theory that *TAC*3 expression could be up-regulated in response to poor placental perfusion. However, it is still not known at what stage in pregnancy these differences first occur. In spite of this, term placental cytotrophoblasts grown in hypoxic conditions were found to be associated with decreases in *TAC*3 expression, whilst cytotrophoblasts exposed to conditions of oxidative stress demonstrated no significant changes in *TAC*3 expression from controls [34]. These data do not appear to support a role for hypoxia or oxidative stress as an underlying cause of the elevated NKB levels in pre-eclampsia. Regardless, it was noted that the outcome was similar to that found for activin A in cytotrophoblasts whose gene is likewise down-regulated by hypoxia, but associated with higher circulating levels of the protein during pre-eclampsia [35]. In another respect, it is also of interest to note that Hoang et al. [36] showed that trophoblast hypoxia induced several proteins that were also found to be targets of NKB suppression [37]. This lead Sawicki et al. [37] to hypothesize that excessive NKB could be thus inhibiting a normal cellular response to hypoxia.

In the rat, where a longitudinal study of *TAC*3 (which has 80% sequence identity with human *TAC*3) expression could be undertaken during late pregnancy there was found to be a significant three fold decrease in *TAC*3 expression from day 16 to 21 [34]. It is not known whether there is a similar decrease in human *TAC*3 expression or whether this represents a species-specific difference. Moreover, by day 19, the expression of the placental rat NK3 receptor (*TACR*3) appeared to be virtually non-existent indicating the gene was switched off [34]. In essence, it was concluded that in normal late pregnancy, at least in the rat, there is a down regulation of the NKB/NK3 ligand-receptor pair in the placenta [34]; a similar down regulation has also been reported in the rat uterus [38,39]. A prime candidate possibly responsible for the down regulation of the NKB/NK3 ligand-receptor pair in late pregnancy is placental estrogen [34]; where its synthesis occurs exponentially with the highest serum levels reached in late pregnancy [38]. Furthermore, computer analysis of the human *TAC*3 gene promoter has revealed the presence of estrogen response elements and many potential half sites [13]. Cintado et al. [40] tentatively suggested that the NKB/NK3 pair could be involved in or, at least be, an indicator of estrogen-related pathophysiologies. Indeed, low circulating levels of estrogen have been associated with pre-eclampsia [41]. Therefore, in the case of pre-eclampsia, possible mechanisms of high NKB expression could include the disruption/failure to down-regulate the NKB/NK3 system during the third trimester as observed in the rat, or represent an unknown stimulus inducing NKB expression [34].

Genomic analysis has also been performed to determine whether the altered circulating NKB levels are due to sequence variants in the encoding *TAC*3 and *TACR*3 genes [42]. Mutations were identified in the promoter, intronic and exonic regions but no association could be demonstrated between NKB level range and any particular genotype or haplotype in the pre-eclamptic or control groups [42]. On the whole the lack of a clear relationship between any polymorphism and circulating NKB has suggested that regulation of these genes occurs at another level other than transcription [42]. Indeed, we had previously noted that many studies had reported higher fold levels of circulating NKB peptide than have been reported in mRNA placental expression levels [34]. Therefore, it is likely that circulating levels of NKB will be determined by many other factors including the manner of processing, turnover, storage, secretion and degradation which are not reflective of mRNA levels. Adding weight to this proposition is the fact that we found very little, if any, processed NKB in normal term placenta [24,27]. Instead the predominant NKB-like immunoreactivity was found in peaks of much larger molecular mass. A similar observation has also been reported by Lowry [43] who observed that the placental concentrations of processed NKB peptides from normal pregnancies were very low and extracts contained more partially processed material than fully processed products. This was also evident in the maternal blood where NKB precursor peptides could also be found [43]. In contrast the chromatographic profiles from term pre-eclamptic placenta had a single peak that contained significant amounts of processed NKB that corresponded to that of NKB found in the rat brain and to that of synthetic NKB [24,27]. This would indicate that although changes are seen at the mRNA expression level the most striking differences are those seen at the level of post-translational processing. It has been proposed that these differences occur as a possible result of differential processing (or lack of during normal pregnancy) at the NH_2_-terminal dibasic cleavage site of NKB [24,27]. This could be the result of increased activity of certain prohormone convertases during pre-eclampsia [27]. Indeed, there is evidence to support the fact that many peptides are not fully processed in the normal placenta but undergo processing to active peptides during pre-eclampsia [44,45]. This could represent part of the mechanism that leads to the increased circulating levels of NKB during pre-eclampsia and could go some way to explaining the differences of NKB concentrations in maternal blood [24].

## What is the physiological significance of NKB in the placenta?

The physiological roles of NKB in the placenta are only just starting to unravel themselves, but one thing is clear the expression levels of *TAC*3 in the placenta are considerably higher than in any other organ or tissue in the human body so far analysed [11,34]. Traditionally, classified as a neurotransmitter placental NKB expression levels are found to be 2.6 fold higher than that in the brain [24] and since, the placenta does not have any neuronal connections; its main action here is proposed to be endocrine rather than neuronal. Several studies have substantiated that the main site of this expression is the outer syncytiotrophoblasts [6,22,23,31], a layer of cells, which are in an ideal position to secrete NKB into the bloodstream. From here, NKB has been demonstrated not only to enter the maternal blood circulation but also to enter the fetal circulation [6,20,25]. Furthermore, significantly higher levels of NKB have been reported in the umbilical cord of pre-eclamptic women when compared to normotensive pregnant women [20]. It is most likely that the secretion of NKB from the placenta is continuous being by the constitutive pathway opposed to being stored in secretory granules (as in neuronal tissue). This may account for the fact that even though placental *TAC*3 mRNA levels are higher than those found in the brain typically NKB peptide levels found in pre-eclamptic placenta are three times lower than those detected in rat brain [24]. In addition, for a tachykinin peptide, such as NKB, to possess agonist activity it is important for it to have undergone COOH-terminal amidation [46]. This can only happen if the precursor protein from which NKB arises is fully processed. There is evidence to support the fact that very little processing of NKB actually occurs in the normal placenta [24,27,43]. Indeed, it appears that most NKB is fully processed only in the pre-eclamptic placenta [24,27] and therefore the condition somehow represents a switch to the production of active NKB. This raises questions regarding the physiological significance of NKB during normal pregnancy as it is unclear how much active peptide is being produced to be secreted. However, it is clear that even in early normal pregnancy processed NKB can start to enter the maternal bloodstream at varying concentrations in different women [6,22,29].

The biological effect of NKB, for example, constriction or dilation of blood vessels depends on the relative tissue distribution of, and binding to, the three tachykinin receptor subtypes given here in order of affinity for NKB, NK3>NK2>NK1 [9]. More recently, we identified the molecular mechanism that leads to the formation of a truncated form of the NK1 receptor, which we have found to be predominantly expressed in peripheral tissue including the placenta where it is proposed to have slightly different pharmacological properties from the full length NK1 receptor [10]. A fourth NK4 receptor had also been cloned from a human placental cDNA library [47]. After subsequent investigation we unfortunately found this receptor to be the guinea pig NK3 receptor [48]. Our first indication of a physiological role for NKB in the utero-placental unit came following our observation of a 37% gain in weight of the uteri of rats infused with chronic doses of NKB providing indirect evidence for an increase in blood supply to the uterus [6]. Thus, we proposed that activation by NKB might play a role in local nitric oxide (NO) release, where NO production by the vascular endothelium of the utero-placental circulation has been well documented in humans [13]. D'Anna et al. [18] has shown a significant correlation between pregnancies complicated with pre-eclampsia and IUGR, increased NKB plasma levels and increased NO metabolite levels. In addition, our original hypothesis suggested that following reduced placental perfusion, NKB would be secreted, to compensate, by increasing blood flow to the fetal-placental unit. This scenario has been supported by observations using perfused human placental cotyledons [49] and placental stem villous arteries [50]. These studies used preconstruction with U-46619 to show that NKB acted as a dilator in the placental vasculature [49,50], where this effect was found to be initiated solely though the NK1 receptor. The NK1 receptor is associated with vasodilatory responses [6], and evidence has shown that the vasoconstrictive NK3 receptor is either absent or expressed at extremely low levels in the human placenta at term when compared to the NK1 and NK2 receptors [34,49]. This would advocate a mechanism in the human placenta, whereby high NKB levels induce placental vasodilatation via the NK1 receptor to maintain low placental resistance. However, no evidence for the involvement of either NO or prostacyclin in this response was found by Brownbill et al. [49]. Laliberte et al. [50] found that removal of the endothelium from the villous arteries did not alter the vasodilatory response concluding that NKB caused an endothelium-independent relaxation of the placental arterial vessels. Nonetheless, pre-eclampsia is typically associated with maternal vasoconstrictive responses and we proposed that activation of NK3 receptors on the venous side of the maternal circulation could be responsible for the hypertension that develops during pre-eclampsia [6,11]. The observation of NK3 receptors in the circulatory system had remained an enigma up to this time [11]. In rats, our original experiments demonstrated that high doses of NKB caused a significant, albeit transient, rise in mean arterial blood pressure [6]. Yang et al. [51] have gone further to analysis this response following the chronic infusion of NKB into female rats finding the cardiovascular responses to be both dose-dependent and bimodal. Low dose NKB was found to reduce resting systemic blood pressure, and reduce vascular reactivity to phenylephrine *in vivo *and in *vitro*. By contrast, high dose NKB increased resting blood pressure, increased the pressor response to angiotensin II *in vivo *and increased maximum phenylephrine induced vasoconstriction *in vitro*. The effects of low dose NKB were shown to be through the direct modulation of vascular tone, while chronic high dose NKB induced increases in blood pressure through both central and peripheral pathways [51]. This would appear to support a mechanism whereby in normal pregnancy NKB may contribute to the modest fall in systemic blood pressure and reduced vascular tone, however high levels may contribute to raising blood pressure and increasing the pressor sensitivity to angiotensin II as seen in pre-eclampsia. However, this rationale has not received universal acceptance and Wareing et al. [52] concluded that NKB was not a circulating factor associated with increase vascular resistance as it did not in their isolated vessel system alter vasoactive responsiveness. On the other hand, Yang et al. [51] stress that treatment of intact animals with high dose NKB more closely mimics the situation of pre-eclampsia than acute incubation of blood vessels *in vitro*.

Beyond the hypertension, the discovery of secreted NKB during pre-eclampsia has led other researchers to investigate some of the other associated symptoms of pre-eclampsia. In severe cases of pre-eclampsia, we predicted that concentrations of NKB may be high enough to stimulate peripheral NK1 receptors on platelets. Since, a major complication of pre-eclampsia is platelet pathology; Graham et al. [14] examined a potential peripheral role for tachykinins in the regulation of platelet function. They reported the expression of NK1 and NK3 receptors on human platelets, and found that the level of activation of platelets by the tachykinin substance P is reduced in NK1 receptor-deficient mouse platelets. Subsequently, it has been demonstrated that inhibition of the NK1 receptor results in substantially reduced thrombus formation *in vitro *under arterial flow conditions, increased bleeding times in mice, and a decrease in experimentally induced thromboembolism [53]. This has lead to the proposal that tachykinins may play a key role in the positive feedback regulation of platelet aggregation and thrombus formation and may offer an alternative therapeutic target to those currently available. The significance of the NK3 receptor on platelets is currently unknown. In another facet, multi-organ oedema is one of the signs of pre-eclampsia, and acute pulmonary oedema is a major cause of death in those affected by pre-eclampsia [54]. Substance P is a well-established peripheral pro-inflammatory peptide and is a potent mediator of increased microvascular permeability, leading to plasma extravasation and tissue oedema formation. Until, fairly recently, a possible role for NKB in peripheral pathologies had not received much attention as it had never before been found in the periphery [6]. Data now suggests that intravenous administration of NKB into wild-type mice can produce significant plasma extravasation in skin, uterus, liver and particularly in the lung [16]. Unexpectedly, the results also indicated a novel NK receptor independent pathway specific to NKB that operates in the mouse lung. Grant et al. [16] concluded that their data was in keeping with a role for NKB in mediating plasma extravasation in diseases such as pre-eclampsia. Nonetheless, the most dangerous complication of pre-eclampsia is the generalized tonic/clonic convulsions of eclampsia that affects the whole brain. Wasseff [17] has hypothesized that it is the effect of elevated NKB that may trigger the convulsions both, directly by enhancing glutamatergic pathways, and indirectly through endothelial activation and endothelin release. Wasseff [17] concludes that these findings strongly suggest that NKB plays a role in altering neuronal excitability in eclampsia. Nonetheless, this is a complex area with several factors also implicated such as proinflammatory cytokines with parallels also drawn between the seizures of eclampsia and those of epilepsy [17].

There is also evidence to support an autocrine/paracrine role for NKB in the placenta. Female rats treated with the NK3 receptor antagonist SR142801 before mating exhibited a tendency towards decreased fertility with a significant reduction in their litter sizes [55]. In addition, the highest levels of uterine NK3 receptor expression in the pregnant rat (peaking at day 3) are detected before and at the time of implantation [38]. NKB could therefore play a key role in modulating implantation in early pregnancy. Indeed, in humans, elevated levels have also been associated with IUGR that like pre-eclampsia occurs with impaired placental implantation and the early failure of trophoblast invasion may represent a time of sustained NKB synthesis [34]. Evidence from direct treatment of term villous cytotrophoblasts with NKB also finds the suppression of several proteins important in the cellular response to oxidative stress and hypoxia [37], which could compound the placental reaction to NKB. More recently, it has become evident that a key component in regulating trophoblast cell survival and function is an imbalance between circulatory pro-angiogenic and anti-angiogenic factors [1]. The placenta has been shown to be a rich source of these factors. One notable factor, soluble flt-1 (sFLT-1) acts as an anti-angiogenic factor by interacting with, and thereby neutralising the effects of vascular endothelial growth factor (VEGF) and placental growth factor (PIGF) [56] therefore reducing the effects at the VEGF receptor-1 (VEGFR-1) and VEGFR-2 receptors, respectively. Increased blood levels of sFLT-1 are shown to correlate with the severity of pre-eclampsia, whereas, opposing, the quantities of VEGF and PIGF are decreased in women with pre-eclampsia compared to controls. In 2006, Pal et al. [57] reported that NKB was an anti-angiogenic factor that inhibited endothelial cell vascular network assembly and opposed angiogenesis. The functional consequences of this cause a direct down-regulation of VEGFR-1 and VEGFR-2 expression, with a corresponding down regulation of VEGF, decreased cell motility and increased calreticulin synthesis [57]. Therefore, NKB joins a growing list of components that may be responsible for creating an anti-angiogenic environment that leads up to the development of pre-eclampsia. As an aside, when attempting to isolate NKB from placenta Lovell et al. [58] have detected a number of co-purifying substances presumed to be in the molecular range 0.9 - 1.3 kDa (c.f. NKB 1.21 kDa). They speculate that these could be autolytic by-products of NKB and other peptides that contain an attached phosphocholine group, which could play a paracrine role when secreted and exposed at the placental interface in order to reduce anti-placental directed antibodies [58]. Their significance still needs to be verified with Lowry [43] reporting that their characterisation will be difficult because of several reasons including their extremely low concentrations, the lack of a purified preparation that gives a clean mass ion and the availability of good quality placenta.

## Conclusion

The precise etiopathogenesis of pre-eclampsia remains the subject of much debate and intensive research. The last ten years has heralded many advances and revealed potential new markers, yet no defining predictive test is yet available to identify women who will subsequently develop pre-eclampsia. This is likely to reflect the fact that pre-eclampsia is multifactorial with an onset, severity and progression that can be significantly distinct in different women. This raises questions to the presence of factors such as NKB to whether they are causative or merely associated with pre-eclampsia. Yet, there is evidence to support a role for NKB right from the time of implantation to the high levels found circulating in the blood of pre-eclamptic women at term. It is apparent the high levels of circulating NKB are a diagnostic feature of pre-eclampsia at term, but its role as an indicator before the onset of symptoms is less clear. Longitudinal studies are still required along with the development of a user friendly and robust test that can be performed directly using plasma. In terms of understanding the regulation of genes involved in pre-eclampsia, the results with *TAC*3 indicate not only to take into account the possibility of up-regulation, but the potential failure/disruption to down-regulate. The role of hormones such as estrogen in these responses will be interesting to investigate for their effect on gene expression. However, the main mechanism of control appears at the level of post-translational modification and may indicate that a general increased activity of certain processing enzymes occurs during pre-eclampsia. This could go some way to explaining the increased plethora of vasoactive peptides that are secreted during pre-eclampsia and is certainly worthy of further research to determine whether there are common processing pathways involved in the elevation of these vasoactive peptides. NKB in the placenta is found to have both paracrine and endocrine roles. In a paracrine role it may modulate implantation, suppress several proteins involved in the cellular response to hypoxia and oxidative stress and act as an anti-angiogenic factor. Locally, it maintains low placental resistance, while its dose dependent and bimodal effects support a mechanism whereby in normal pregnancy it contributes to the modest fall in blood pressure and reduced vascular tone, whilst at high doses it contributes to raising blood pressure and increasing pressor sensitivity as seen in pre-eclampsia. Animal models also show that in the periphery NKB can have significant effects on platelet clotting and lung oedema. The repetition of animal studies with potent and selective combinations of NK receptor antagonists is needed to show their potential effectiveness in the treatment of pre-eclampsia. NK3 antagonists might alleviate the effects of high NKB in the plasma of mothers suffering from pre-eclampsia, whilst NK1 antagonists could control the effects of thrombocytopenia and generalized inflammation. Moreover, recent research has shown a diversity of mechanisms such as mRNA splicing, precursor processing and post-translation modification that can lead to a complex and continually expanding repertoire of tachykinin peptides. The proteomic analysis of the tachykinins has been hindered by the lack of specific methodologies to capture, purify and characterize them and it will be important in the future to develop these methodologies [59]. We look forward to the next ten years of research in the field of tachykinins and pregnancy!

## Abbreviations

**NKB**: neurokinin B; **TAC3**: tachykinin 3 gene; **RIA**: radioimmunoassay; **ELISA**: enzyme-linked immunosorbant assay; **NK1**: tachykinin 1 receptor; **NK2**: tachykinin 2 receptor; **NK3**: tachykinin 3 receptor; **IUGR**: intrauterine growth restriction; **TACR3**: tachykinin 3 receptor gene; **NO**: nitric oxide; **SFTL-1**: soluble FLT-1; **VEGF**: vascular endothelial growth factor; **PIGF**: placental growth factor; **VEGFR-1**: VEGF receptor-1; **VEGFR-2**: VEGF receptor-2.

## Competing interests

The author is listed as an inventor on patent WO0136979 (placental human neurokinin B precursor).

## Authors' contributions

NP drafted and wrote the manuscript. The author read and approved the final manuscript.
